# Fecal Short-Chain Fatty Acids Levels Were Not Associated With Autism Spectrum Disorders in Chinese Children: A Case–Control Study

**DOI:** 10.3389/fnins.2019.01216

**Published:** 2019-11-29

**Authors:** Jue Wang, Jialiang Pan, Hengying Chen, Yan Li, William Kwame Amakye, Jingjing Liang, Bingjie Ma, Xinwei Chu, Limei Mao, Zheqing Zhang

**Affiliations:** ^1^Department of Nutrition and Food Hygiene, Guangdong Provincial Key Laboratory of Tropical Disease Research, School of Public Health, Southern Medical University, Guangzhou, China; ^2^Qiaodong Community Health Center, Shenzhen Yantian District People’s Hospital, Shenzhen, China; ^3^Department of Hygiene Detection Center, Guangdong Provincial Key Laboratory of Tropical Disease Research, School of Public Health, Southern Medical University, Guangzhou, China; ^4^Department of Child Health Care, Guangzhou Women and Children’s Medical Center, Guangzhou Medical University, Guangzhou, China

**Keywords:** short-chain fatty acids, autism spectrum disorders, case–control study, children, Chinese

## Abstract

Evidence from animal models supports a link between short-chain fatty acids (SCFAs), a key subset of gut microbial metabolites, and autism spectrum disorders (ASD). However, findings from human studies on this topic are unclear. We aimed to investigate whether fecal SCFAs are associated with ASD in Chinese children aged 6–9 years old. A total of 45 ASD children aged 6–9 years and 90 sex- and age-matched neurotypical controls were enrolled. High-performance liquid chromatography was applied to quantify 10 SCFA subtypes in feces. Dietary and other socio-demographic information were obtained via face-to-face interview using questionnaires. After adjustment for multiple comparisons, paired *t*-test analysis indicated that the fecal total and subtype SCFA concentrations were comparable in autistic children and the controls. Conditional logistic regression analysis showed that there was no significant relationship between the fecal concentration of SCFAs and the risk of ASD after adjustment for age, sex, BMI, breastfeeding, mode of delivery, parental education level, and daily energy, protein, fat, and fiber intake. In conclusion, our results did not support the hypothesis that fecal SCFA levels might be associated with the presence of ASD. However, SCFA measurement was based on a single stool sample test, so this conclusion should be treated with caution. Further studies with measurement of long-term bodily SCFA concentrations are needed to examine this relationship.

## Introduction

Autism spectrum disorder (ASD) is a serious neurodevelopmental disorder placing a substantial burden on individuals, families, and society (Lyall et al., 2017). The most recent estimate from the National Health Interview Survey of the United States showed that ASD prevalence was 2.47% among United States children and adolescents in 2014–2016 (Xu et al., 2018). In China, a new meta-analysis based on 44 studies found that the pooled prevalence of ASD was 39.23 per 10000 (95% CI: 28.44–50.03 per 10,000, *I*^2^ = 89.2%) (Wang et al., 2018). ASD is associated with substantial disability and economic loss. A study reported that the average loss of annual income associated with having a child with ASD was Chinese RenMinBi (RMB) 44077 ($7226) compared with RMB 20788 ($3408) for families with some other disabled children (Ou et al., 2015). Therefore, searching for approaches to improving the core deficits and lives of people with ASD is of great importance.

Accumulating evidence demonstrates that gastrointestinal symptoms, such as abdominal pain, gaseousness, diarrhea, constipation, and flatulence, are common comorbidities in patients with ASD (Chaidez et al., 2014). A review article reported that the median prevalence of one or more gastrointestinal symptoms in ASD was 46.8% (Holingue et al., 2018), suggesting that gastrointestinal factors may play an important role in the pathogenesis of ASD and/or related complications. The gut-brain-microbiome axis connecting the gut and the brain through the neuroendocrine, neuroimmune, and autonomic nervous systems and via microbiotic toxin production provides a possible way to affect the brain (Li et al., 2017). The major metabolic products of enteric bacteria following fermentation of dietary carbohydrates and some amino acids are short-chain fatty acids (SCFAs). The most abundant of these are acetic acid, propionic acid, and butyric acid, representing 90–95% of the SCFAs present in the colon.

In rodent models, the administration of propionic acid could produce reversible behavioral changes closely resembling those found in ASD (MacFabe, 2015). Removal of refined carbohydrates, a substrate for enteric bacteria that produce propionic acid from the diet, resulted in improvement in ASD symptoms (MacFabe et al., 2007). Butyrate was also found to improve ASD-type behavior in a mouse model of autism (Takuma et al., 2014). In addition, butyrate appeared to be neuroprotective in lymphoblastoid cell line in boys with autism (Frye et al., 2017; Rose et al., 2018). Al-Owain et al. (2013) also reported the occurrence of autism in a 7-year-old girl with propionic acidemia. The mechanism of the effects of these major SCFAs may be through the alteration of mitochondrial function via the citric acid cycle and carnitine metabolism (Rose et al., 2018), inducing changes in brain phospholipids (Thomas et al., 2010), the epigenetic modulation of ASD-associated genes (Nankova et al., 2014), or modulation of immunological pathways (Frye et al., 2017).

However, findings from previous human studies on the associations between propionate, butyrate, and acetate and ASD have been mixed: fecal concentrations of these SCFAs were shown to be lower (Adams et al., 2011; De Angelis et al., 2013; Liu et al., 2019), higher (Wang et al., 2012; Berding and Donovan, 2018), or comparable (Kang et al., 2018) in children with autism in comparison with healthy controls. Other SCFAs of low concentration were also reported to be inconsistently increased or reduced in different studies (Adams et al., 2011; Wang et al., 2012; Berding and Donovan, 2018; Liu et al., 2019). Multiple factors affect the concentration of SCFAs in feces, including amount/type of fermentable carbohydrate consumption, preservatives in many processed foods, composition/diversity of the microbiota, colonic SCFA absorption, colonic transit time, interaction with other metabolites, and interactions between microbes and the host (den Besten et al., 2013; MacFabe, 2015). The majority of previous studies linking fecal SCFAs and autism have been performed in the United States and Europe. Considering the genetic, dietary, environmental and metabolic differences between Chinese and their western counterparts, it is necessary to evaluate the relationship between fecal SCFAs and ASD in Chinese children.

Until now, only one study has explored this topic. This studied 30 ASD children and 20 neurotypical controls in China, and only focused on propionic, butyric, acetic, and valeric acids (Liu et al., 2019). We sought to conduct a case–control study among 45 ASD children between 6–9 years of age and 90 sex- and age-matched neurotypical children to examine whether total SCFAs and 10 SCFA subtypes were correlated with autism in Chinese children.

## Materials and Methods

### Ethics Statement

The Ethical Committee of the School of Public Health, Sun Yat-sen University approved this study (No. 2018047). The study was performed in accordance with the Helsinki Declaration, as previously described (Ma et al., 2019). The parents or guardians of all subjects provided their written informed consent before the study.

### Subject Recruitment

A case–control study was conducted. Each case was matched by two controls for sex and age. Forty-five children with ASD (40 males and 5 females) aged 6–9 years were recruited from the Center for Child and Adolescent Psychology and Behavioral Development of Sun Yat-sen University in Guangzhou, China, between December 2015 and July 2017. The children were assessed for ASD diagnosis and met the criteria in the Diagnostic and Statistical Manual of Mental Disorders, 5th Edition (DSM-5) (American Psychiatric Association, 2013). Two experienced developmental and behavioral pediatricians further confirmed the diagnosis using the Childhood Autism Rating Scale (CARS) (Schopler et al., 1980). Children with ASD were excluded from the study if they had a history of Rett syndrome and cerebral palsy and other congenital diseases. Ninety age-/sex-matched healthy, developmentally normal children who were unrelated to the autistic subjects in this study were enrolled from primary schools in urban Guangzhou city. Both cases and controls who had a history of celiac disease, inflammatory bowel disease, or antibiotic, probiotic or prebiotic treatment or any other medical treatment within the 3 months prior to the study were excluded, as described elsewhere (Ma et al., 2019). Although some evidence suggests that antibiotic-induced microbiota alterations can remain after long periods of time, spanning months and even years (Jernberg et al., 2010), a recent study suggested that the gut microbiota of subjects recovered to near-baseline composition within 1.5 months (Palleja et al., 2018). Therefore, we used a 3-month cut off in this study.

### Anthropometric Measures

Height was measured to the nearest 0.1 cm and weight to the nearest 0.5 kg, with the subjects wearing light clothing and no shoes. Body mass index (BMI) was calculated as weight (in kg)/height (in m^2^).

### Analysis of Short-Chain Fatty Acids

Fresh fecal matter was collected from each participant and was stored in sterile tubes at −80°C until analysis, without freeze–thaw cycling. Eleven SCFAs, namely acetic, propionic, n-butyric, crotonic, iso-butyric, 2-methylbutyric, isovaleric, valeric, 2-ethylbutyric (internal standard), 4-methylvaleric, and hexanoic acid were detected and quantified via a reverse-phase high-performance liquid chromatography (HPLC) method as reported by Torii et al. (2010) with slight modifications. Fecal matter (about 0.2–0.5 g) was added to 5.0 mL of 70% ethanol in a weighed glass centrifuge tube with a screw cap and centrifuged at 20°C, 2500 rpm for 10 min. 300 μL of the supernatant was transferred into a new tube and mixed with 50 μL of the internal standard and 300 μL each of pyridine (Reagent 1), 1-EDC-HCl (Reagent 2), and 2-NPH-HCl (Reagent 3) solutions as reaction-assistive agents. The mixture was reacted at 60°C for 20 min, mixed with 200 μL of potassium hydroxide solution (Reagent 4) as a reaction stopper, and then further reacted at 60°C for 20 min. After cooling, the mixture was shaken with 3.0 mL of phosphoric acid aqueous solution (Reagent 5) and 4.0 mL of ether for three minutes and centrifuged. The obtained ether layer was shaken with 4.0 mL of water for three minutes and centrifuged. The ether layer was obtained, and ether was eliminated using nitrogen gas. The obtained fatty acid hydrazide was dissolved in 200 μL of methanol, and 30 μL was subjected to HPLC.

The HPLC system was comprised of an Agilent 1200 Hewlett Packard (Santa Clara, CA, United States) HPLC machine. The column was a YMC-Pack FA 250 × 6 mm ID column (YMC, Kyoto, Japan). The mobile phase was composed of acetonitrile–methanol–water at a ratio of 3.3:1.7:94.9, respectively, with a gradual change to 53.4:28.5:18.0 over a 30-min interval and then gradually modified back to the initial ratios and maintained to the end of the analysis of each sample. The pH was adjusted to 4.5 by using 0.1% tri-fluoro acetic acid (TFA: Wako Pure Chemical). The column temperature was kept at 50°C, while the flow rate was set to 1.1 mL/min. The measurement wavelength was 400 nm. A pooled fecal supernatant sample was analyzed with batches of study samples to monitor analytic precision, and the coefficient of variation for the reproducibility test was 2.4–9.8% and 3.9–11.4% for intra-day and inter-day reproducibility, respectively, with a recovery rate of 80–120% for all SCFAs. Considering that the distribution of SCFAs in feces is uneven, each sample was tested in triplicate, and the mean value was calculated. The intra-class correlation coefficient of fecal SCFAs was from 0.797 to 0.967.

### Covariates

A 3-day food diary (2 ordinary school days and 1 weekend day) and a validated 79-item food frequency questionnaire (FFQ) were used to assess the subjects’ habitual dietary intakes via face-to-face interviews by trained interviewers (Zhang and Ho, 2009). For each item of FFQ, subjects were required to recall the frequency (never, or per year, month, week, or day) and approximate portion sizes they had consumed during the previous 12 months. Color pictures of foods in standard sizes were provided to help attenuate recall bias, and photographs of food portion sizes were also provided to help estimate the amount of food consumed. Parents and their children were asked to respond to the questionnaires together. Daily mean nutrient and energy intakes were calculated using the Chinese Food Composition Table, 2009 (Yang, 2009). The average values of the FFQ and 3-day food diaries were used for further analysis. Individual daily intake of each nutrient was adjusted for total energy intake by using the regression-residual method (Willett et al., 1997). Essential information including age, height, weight, delivery mode, feeding patterns during infancy, and parents’ educational level was also collected. We classified parental education level into four categories: secondary or less, graduate, and postgraduate or above.

### Statistical Analysis

Continuous variables were presented as means ± standard deviation, and comparisons between ASD and neurotypical groups were performed with paired sample *t*-tests. Categorical variables were presented as proportions, and the group differences were compared using chi-square tests. We grouped total SCFAs and SCFA subclasses into tertiles based on control subjects (Turner et al., 2010) (Supplementary Data Sheet S1). The lowest tertile served as the reference group. We used univariate and multivariate conditional logistic regression to assess the association between SCFAs and the risk of autism, and the associations were expressed as odds ratios (ORs) with 95% confidence intervals (CIs). In the multivariate analyses, adjustments were made for age, sex, BMI, energy intake, protein, fat and fiber intake, mode of delivery, breastfeeding, and parental education level. Potential confounders were identified by drawing a directed acyclic graph (DAG) using DAGitty version 2.3 (Textor et al., 2011). Dietary fiber and protein intake were adjusted because they are the main substrate for SCFA production and are associated with autism. Similarly, adjustment for caloric intake was justified because individuals might have higher SCFA levels because, on the whole, they ate more, which can contribute to autism. The other covariates included in the multivariable models also constitute potential confounders because of their association with autism and SCFA production (Supplementary Figure S1). Considering the type I error caused by multiple testing, *P*-values were adjusted by using Bonferroni correction, and the statistical threshold was adjusted to 0.05/11 = 0.0045 (Armstrong, 2014). All statistical procedures were carried out with SPSS^®^ for Windows software (version 22.0, SPSS Inc., Chicago, IL, United States).

## Results

### Participant Characteristics

We recruited 45 subjects with ASD (40 males and 5 females) and 90 sex- and age-matched neurotypical controls. Table 1 shows the characteristics of the subjects. Children in the autism group were younger than their neurotypical controls (7.67 ± 1.17 vs. 7.73 ± 1.13 years, *P* = 0.008). No significant group difference was found in terms of the other characteristics.

**TABLE 1:** Characteristics of study participants.

**Variables**	**ASD**	**Control**	***P*^∗^**
			
	**Mean ± SD**	**Mean ± SD**	
Age (years)	7.67 ± 1.17	7.73 ± 1.13	0.008
Sex			
Male	40	80	
Female	5	10	
Height (cm)	126.8 ± 8.23	127.3 ± 7.68	0.594
Weight (kg)	27.0 ± 7.22	26.9 ± 7.39	0.825
BMI (kg/m^2^)	16.6 ± 3.13	16.4 ± 2.88	0.406
Delivery mode			0.714
Vaginal	20 (44.4)	43 (47.8)	
Cesarean	25 (55.6)	47 (52.2)	
Feeding patterns			0.131
Breastfeeding	35 (77.8)	79 (87.8)	
Artificial feeding	10 (22.2)	11 (12.2)	
Maternal education			0.231
Secondary or less	21 (46.7)	32 (35.6)	
University	23 (51.1)	48 (53.3)	
Postgraduate or above	1 (2.2)	8 (8.9)	
Paternal education			0.730
Secondary or less	17 (37.8)	39 (43.3)	
University	23 (51.1)	44 (48.9)	
Postgraduate or above	5 (11.1)	7 (7.8)	
Dietary status			
Total energy (kcal/d)	1474 ± 347	1444 ± 222	0.531
Carbohydrate^a^ (g/d)	214 ± 25	206 ± 19	0.092
Fat^a^ (g/d)	41.0 ± 8.55	42.7 ± 6.01	0.274
Protein^a^ (g/d)	64.5 ± 11.0	67.7 ± 7.86	0.093
Fiber ^a^ (g/d)	8.59 ± 3.16	8.29 ± 2.41	0.614

*ASD, autism spectrum disorders. ^*a*^ Energy-adjusted by the residual method. ^∗^The *P*-value was compared between ASD and control groups.*

### Fecal Short-Chain Fatty Acids

Table 2 presents the paired *t*-test results between cases and controls for the SCFAs. The concentrations of fecal total SCFA, acetate, propionate, and butyrate were 111.7 ± 42.2, 56.3 ± 21.1, 25.0 ± 12.6, and 22.7 ± 12.5 μmol/g in autistic children and 106.2 ± 29.2, 53.1 ± 16.6, 22.8 ± 7.87, and 21.4 ± 7.58 μmol/g in controls, respectively. The percentages of acetate, propionate, and butyrate were 50.8, 22.2, and 19.9% in the autism group and 49.9, 21.4, and 19.9% in the control group, respectively. However, the differences were not statistically significant (*P* > 0.0045). Other SCFA subtypes accounted for less than 10% of SCFAs in feces and were also not significantly different between groups.

**TABLE 2:** Concentrations of SCFAs among control and ASD groups.

**Variables**	**ASD**		**Control**		***P***
			
	**Mean**	**SD**	**Mean**	**SD**	
Acetic acid (μmol/g)	56.3	21.1	53.1	16.6	0.459
Acetic acid percentage (%)	50.8	8.02	49.9	5.07	0.519
Propionic acid (μmol/g)	25.0	12.6	22.8	7.87	0.346
Propionic acid percentage (%)	22.2	5.81	21.4	4.32	0.494
n-Butyric acid (μmol/g)	22.7	12.5	21.4	7.58	0.582
n-Butyric acid (%)	19.9	6.14	19.9	3.41	0.985
Crotonic acid (μmol/g)	0.15	0.22	0.28	0.36	0.053
Crotonic acid Percentage (%)	0.13	0.17	0.29	0.36	0.013
Isobutyric acid (μmol/g)	1.87	1.07	2.09	0.77	0.191
Isobutyric acid (%)	1.77	0.98	2.08	0.74	0.088
2-Methylbutyric acid (μmol/g)	0.98	0.67	1.23	0.53	0.015
2-Methylbutyric acid (%)	0.93	0.63	1.24	0.54	0.011
Iso-Valeric acid (μmol/g)	1.68	1.22	2.02	0.93	0.080
Iso-Valeric acid (%)	1.58	1.13	1.99	0.82	0.043
Valeric acid (μmol/g)	2.30	2.15	2.57	1.47	0.398
Valeric acid percentage (%)	2.07	1.41	2.47	1.29	0.106
4-Methylvaleric acid (μmol/g)	0.06	0.07	0.13	0.21	0.033
4-Methylvaleric acid percentage (%)	0.05	0.06	0.12	0.19	0.043
Hexanoic acid (μmol/g)	0.66	1.26	0.64	1.05	0.912
Hexanoic acid percentage (%)	0.70	1.35	0.60	0.91	0.663
TSCFAs (μmol/g)	111.7	42.2	106.2	29.2	0.509

*ASD, autism spectrum disorders; SCFAs, short-chain fatty acids; TSCFAs, total short-chain fatty acids.*

### Risk of Autism According to SCFA Levels

The results of the conditional logistic regression analysis are shown in Tables 3, 4. After adjustment for potential confounders, the ORs (95% CI) in the top versus bottom tertiles were 1.17 (0.38, 3.63) for total SCFA, 2.95 (0.85, 10.2) for acetate, 1.21 (0.47, 3.13) for propionate, and 0.87 (0.28, 2.76) for butyrate. Similar results were obtained for the percentages of individual SCFAs. With regards to other SCFA subtypes, no significant relationship with the presence of autism was observed.

**TABLE 3:** ORs and 95% CI of risk of autism according to levels of total SCFAs and SCFA subclasses.

**SCFAs**	**Tertile 1**	**Tertile 2**	**Tertile 3**
**Acetic acid (μmol/g)**	≤40.4	40.4–57.2	≥57.2
Case/Control	10/30	14/30	21/30
OR I (95%CI)^a^	1	1.40 (0.53–3.69)	2.10 (0.84–5.25)
*P*	1	0.497	0.112
OR II (95%CI)^b^	1	1.33 (0.39–4.50)	2.95 (0.85–10.2)
*P*	1	0.645	0.088
**Propionic acid (μmol/g)**	≤17.5	17.5–24.8	≥24.8
Case/Control	12/30	12/30	21/30
OR I (95%CI)^a^	1	0.97 (0.38–2.47)	1.62 (0.72–3.64)
*P*	1	0.943	0.245
OR II (95%CI)^b^	1	0.88 (0.28–2.83)	1.21 (0.47–3.13)
*P*	1	0.832	0.694
**n-Butyric acid (μmol/g)**	≤15.5	15.5–24.5	≥24.5
Case/Control	13/30	18/30	14/30
OR I (95%CI)^a^	1	1.36 (0.58–3.17)	1.05 (0.42–2.63)
*P*	1	0.475	0.913
OR II (95%CI)^b^	1	1.28 (0.46–3.57)	0.87 (0.28–2.76)
*P*	1	0.640	0.818
**Crotonic acid (μmol/g)**	≤0.02	0.02–0.19	≥0.19
Case/Control	17/30	16/30	12/30
OR I (95%CI)^a^	1	0.97 (0.40–2.38)	0.72 (0.30–1.72)
*P*	1	0.944	0.457
OR II (95%CI)^b^	1	1.21 (0.38–3.83)	0.95 (0.33–2.78)
*P*	1	0.752	0.931
**Iso-butyric acid (μmol/g)**	≤1.54	1.54–2.46	≥2.46
Case/Control	21/30	13/30	11/30
OR I (95%CI)^a^	1	0.66 (0.29–1.51)	0.51 (0.20–1.30)
*P*	1	0.328	0.159
OR II (95%CI)^b^	1	0.59 (0.19–1.83)	0.40 (0.12–1.31)
*P*	1	0.364	0.128
**2-Methylbutyric acid (μmol/g)**	≤0.78	0.78–1.45	≥1.45
Case/Control	25/30	9/30	11/30
OR I (95%CI)^a^	1	0.36 (0.14–0.94)	0.45 (0.18–1.10)
*P*	1	0.037	0.081
OR II (95%CI)^b^	1	0.26 (0.07–0.99)	0.33 (0.10–1.07)
*P*	1	0.047	0.065
**Iso-valeric acid (μmol/g)**	≤1.19	1.19–2.38	≥2.38
Case/Control	20/30	15/30	10/30
OR I (95%CI)^a^	1	0.76 (0.34–1.72)	0.49 (0.19–1.25)
*P*	1	0.508	0.133
OR II (95%CI)^b^	1	0.72 (0.22–2.37)	0.46 (0.15–1.41)
*P*	1	0.591	0.175
**Valeric acid (μmol/g)**	≤1.49	1.49–3.01	≥3.01
Case/Control	17/30	16/30	12/30
OR I (95%CI)^a^	1	0.93 (0.39–2.18)	0.69 (0.27–1.75)
*P*	1	0.858	0.433
OR II (95%CI)^b^	1	0.72 (0.24–2.15)	0.47 (0.14–1.59)
*P*	1	0.554	0.222
**4-Methylvaleric acid (μmol/g)**	≤0.02	0.02–0.08	≥0.08
Case/Control	16/30	16/30	13/30
OR I (95%CI)^a^	1	1.00 (0.42–2.36)	0.80 (0.32–2.01)
*P*	1	0.999	0.638
OR II (95%CI)^b^	1	1.31 (0.40–4.27)	1.06 (0.31–3.60)
*P*	1	0.656	0.925
**Hexanoic acid (μmol/g)**	≤0.11	0.11–0.35	≥0.35
Case/Control	17/30	14/30	14/30
OR I (95%CI)^a^	1	0.80 (0.31–2.02)	0.80 (0.32–2.00)
*P*	1	0.630	0.631
OR II (95%CI)^b^	1	0.62 (0.19–1.98)	1.25 (0.39–3.97)
*P*	1	0.416	0.705
**TSCFAs (μmol/g)**	≤86.2	86.2–114.9	≥114.9
Case/Control	13/30	15/30	17/30
OR I (95%CI)^a^	1	1.14 (0.49–2.67)	1.30 (0.54–3.09)
*P*	1	0.766	0.558
OR II (95%CI)^b^	1	0.59 (0.17–1.99)	1.17 (0.38–3.63)
*P*	1	0.395	0.789

*SCFAs, short-chain fatty acids; TSCFAs, total short-chain fatty acids. OR, odds ratio. ^*a*^Non-adjusted (crude) model. ^*b*^Fully adjusted model, adjusted for age, sex, BMI, energy, fiber, protein and fat intake, breastfeeding, delivery mode, and parental education level in the fully adjusted model.*

**TABLE 4:** ORs and 95% CI of risk of autism according to percentages of total SCFAs and SCFA subclasses.

**SCFAs**	**Tertile 1**	**Tertile 2**	**Tertile 3**
**Acetic acid (%)**	≤46.8	46.8–51.6	≥51.6
Case/Control	14/30	9/30	22/30
OR I (95%CI)^a^	1	0.68 (0.27–1.73)	1.65 (0.70–3.91)
*P*	1	0.414	0.252
OR II (95%CI)^b^	1	0.75 (0.23–2.46)	2.21 (0.66–7.42)
*P*	1	0.630	0.201
**Propionic acid (%)**	≤18.5	18.5–23.5	≥23.5
Case/Control	14/30	14/30	17/30
OR I (95%CI)^a^	1	0.99 (0.40–2.45)	1.24 (0.50–3.08)
*P*	1	0.990	0.637
OR II (95%CI)^b^	1	0.73 (0.21–2.54)	1.09 (0.37–3.19)
*P*	1	0.618	0.875
**n-Butyric acid (%)**	≤17.4	17.4–21.4	≥ 21.4
Case/Control	16/30	17/30	12/30
OR I (95%CI)^a^	1	1.02 (0.45–2.32)	0.75 (0.30–1.89)
*P*	1	0.956	0.541
OR II (95%CI)^b^	1	0.80 (0.29–2.16)	0.50 (0.15–1.64)
*P*	1	0.655	0.251
**Crotonic Acid (%)**	≤0.02	0.02–0.19	≥ 0.19
Case/Control	16/30	17/30	12/30
OR I (95%CI)^a^	1	1.10 (0.44–2.79)	0.75 (0.31–1.86)
*P*	1	0.835	0.540
OR II (95%CI)^b^	1	0.96 (0.31–2.99)	0.90 (0.30–2.73)
*P*	1	0.942	0.851
**Iso-butyric Acid (%)**	≤1.53	1.53–2.37	≥ 2.37
Case/Control	21/30	12/30	12/30
OR I (95%CI)^a^	1	0.55 (0.22–1.37)	0.56 (0.23–1.39)
*P*	1	0.200	0.211
OR II (95%CI)^b^	1	0.50 (0.16–1.59)	0.40 (0.12–1.32)
*P*	1	0.240	0.132
**2-Methylbutyric acid (%)**	≤0.83	0.83–1.41	≥ 1.41
Case/Control	25/30	10/30	10/30
OR I (95%CI)^a^	1	0.41 (0.17–0.99)	0.40 (0.16–0.99)
*P*	1	0.047	0.047
OR II (95%CI)^b^	1	0.37 (0.12–1.12)	0.25 (0.07–0.89)
P	1	0.077	0.033
**Iso-valeric acid (%)**	≤1.23	1.23–2.35	≥ 2.35
Case/Control	22/30	14/30	9/30
OR I (95%CI)^a^	1	0.65 (0.28–1.51)	0.41 (0.16–1.06)
*P*	1	0.312	0.066
OR II (95%CI)^b^	1	0.58 (0.18–1.81)	0.26 (0.07–0.96)
*P*	1	0.345	0.043
**Valeric acid (%)**	≤1.56	1.56–3.19	≥ 3.19
Case/Control	18/30	15/30	12/30
OR I (95%CI)^a^	1	0.82 (0.35–1.92)	0.63 (0.24–1.64)
*P*	1	0.642	0.341
OR II (95%CI)^b^	1	0.62 (0.21–1.79)	0.46 (0.13–1.61)
*P*	1	0.377	0.226
**4-Methylvaleric acid (%)**	≤0.02	0.02–0.09	≥ 0.09
Case/Control	19/30	13/30	13/30
OR I (95%CI)^a^	1	0.69 (0.30–1.63)	0.68 (0.28–1.66)
*P*	1	0.402	0.396
OR II (95%CI)^b^	1	0.74 (0.24–2.31)	0.80 (0.26–2.48)
*P*	1	0.609	0.700
**Hexanoic acid (%)**	≤0.12	0.12–0.32	≥ 0.32
Case/Control	17/30	15/30	13/30
OR I (95%CI)^a^	1	0.87 (0.36–2.06)	0.73 (0.28–1.91)
*P*	1	0.744	0.520
OR II (95%CI)^b^	1	0.83 (0.27–2.54)	1.19 (0.36–3.90)
*P*	1	0.740	0.775

*SCFAs, short-chain fatty acids; OR, odds ratio. ^*a*^Non-adjusted (crude) model. ^*b*^Fully adjusted model, adjusted for age, sex, BMI, energy, fiber, protein and fat intake, breastfeeding, delivery mode, and parental education level in the fully adjusted model.*

## Discussion

Based on the data from 45 autistic cases and 90 sex- and age-matched controls, we examined the differences in total SCFAs and the 10 SCFA subclasses in Chinese children. After controlling for potential covariates, no significant associations were observed between fecal total or subclasses of SCFAs and the risk of autism in children.

The results of previous studies on fecal SCFAs and autism are inconsistent. In the study of Kang et al. (2018), no significant group differences in fecal concentration of acetate (158 vs. 162 μmol/g), butyrate (32.6 vs. 37.2 μmol/g), and propionate (38.8 vs. 39.2 μmol/g) were found between 21 children with ASD and 23 neurotypical children between 4 and 17 years old in the United States (*P* > 0.05). However, based on 23 autistic and 31 healthy control children (comprising 22 normal developing siblings of ASD participants and nine community controls without a family history of ASD) aged 3.1 to 18.4 years old in Australia, Wang et al. (2012) found elevated fecal acetic (74.8 vs. 61.7 μmol/g, *P* = 0.037), propionic (24.7 vs. 19.7 μmol/g, *P* = 0.007), butyric (24.5 vs. 19.5 μmol/g, *P* = 0.025), isobutyric (3.1 vs. 2.5 μmol/g, *P* = 0.022), valeric (3.6 vs. 2.8 μmol/g, *P* = 0.004), and isovaleric (4.7 vs. 3.9 μmol/g, *P* = 0.038) acid in the ASD group compared with the controls. Berding and Donovan (2018) reported higher concentrations of acetate (180 vs. 135 μmol/g, *P* = 0.02), propionate (58 vs. 45 μmol/g, *P* = 0.04), and butyrate (58 vs. 40 μmol/g, *P* = 0.03), but no statistically significant differences in the concentrations of valerate, isovalerate, and isobutyrate in the fecal samples of 26 ASD children compared with 32 controls between 2 and 7 years of age in the United States. In contrast, decreased fecal acetate (384 vs. 520 μg/mL, *P* < 0.001), propionate (143 vs. 164 μg/mL, *P* = 0.09), valerate (240 vs. 360 μg/mL, *P* = 0.05), and total SCFA (750 vs. 920 μg/mL, *P* = 0.006) and comparable butyrate were found in 38 ASD children compared with healthy controls aged 2.5–18 years, also in the United States (−18%∼−39%, *P* < 0.01) by Adams et al. (2011). De Angelis et al. (2013) also reported significantly lower levels of total SCFA (3.27 vs. 4.41 ppm, *P* < 0.05) in 10 autistic children aged 4–10 years old. In the study of Liu et al. (2019), lower levels of fecal acetic acid (732.4 vs. 899.9 μg/mL, *P* = 0.011) and butyrate (413.2 vs. 563.3 μg/mL, *P* = 0.005) but a higher level of fecal valeric acid (1646.9 vs. 615.8 μg/mL, *P* < 0.001) were reported to exist in 30 ASD subjects in China with mean age of 4.4 years.

In accordance with the findings from Kang et al. (2018), no significant association between SCFA subtypes or total SCFAs and autism was observed in the present data. In comparison with previous studies, the cases and controls in our research had similar lifestyle characteristics and were matched for sex and age in a narrow age range. In addition, this study had a larger sample size than most of the previous reports, which usually involved fewer than 30 participants in each group (Wang et al., 2012; De Angelis et al., 2013; Berding and Donovan, 2018; Kang et al., 2018) and could thus provide more robust conclusions. Other factors that might also contribute to the observed heterogeneity in study results include differences in the diagnostic criteria (e.g., the Autism Diagnostic Interview-Revised, Autism Diagnostic Observation Schedule, CARS, Diagnostic and Statistical Manual of Mental Disorders, 4th Edition), autism severity, and the methods used to quantify SCFAs (e.g., gas chromatograph, gas chromatography-flame ionization detector). SCFAs are the end products of fermentation of dietary fibers and some amino acids by the anaerobic intestinal microbiota. Differences in microbiota composition and dietary background and their interactions may also contribute to wide differences in results among studies (den Besten et al., 2013). A recent study using bidirectional Mendelian randomization analyses found that host-genetic-driven increase in gut production of butyrate was associated with improved metabolic traits (Sanna et al., 2019). Therefore, genetic background might be another potential reason for the discrepancies between studies.

In autism rat models, repeated (5–14-day) intraventricular administration of propionic acid (0.026, 0.052, 0.26 mol/L, 4 ml, pH buffered to 7.5), approximating the levels found in propionic acidemia patients, induced behaviors resembling ASD and reproduced the neuropathological changes associated with ASD (MacFabe et al., 2007, 2011; Shultz et al., 2008). Propionate has many proposed bioactive effects on neurotransmitter systems, intracellular acidification/calcium release, fatty acid metabolism, gap junction gating, immune function, and epigenetic alteration (MacFabe, 2015). In contrast, in a mouse model of autism, Takuma et al. (2014) found that intraperitoneally injected sodium butyrate (1200 mg/kg/d) attenuated novel object recognition deficits and hippocampal dendritic spine loss. Kratsman et al. (2016) also reported that sodium butyrate (100 mg/kg/d) attenuated social behavior deficits and modified the transcription of inhibitory/excitatory genes in the frontal cortex in an autism model. Findings from the study of Rose et al. (2018) suggested that 1 mmol/L butyrate enhanced mitochondrial function during oxidative stress in lymphoblastoid cell line. Frye et al. (2017) also reported that propionic acid enteric microbiome metabolite PPA could evoke a typical immune activation in LCLs with an underlying abnormal metabolic state. However, the circulating levels of SCFAs in the human central nervous system might be far lower than the dosages used in animal models or cell cultures. Traditionally, hepatic clearance is thought to reduce the systemic effects of SCFAs on systemic metabolic and regulatory pathways. Bloemen et al. (2009) measured the arterial, portal, and hepatic venous concentrations of acetate, propionate, and butyrate in patients undergoing major upper abdominal surgery, and the results indicated that no intestinally produced butyrate and propionate escaped the splanchnic area because of the highly efficient hepatic uptake. However, the distal colon, where the majority of gut microbiome reside, bypasses portal circulation, enabling systemic access (MacFabe, 2015). Propionate in diet as a preservative is absorbed directly in the gastrointestinal tract. A previous study reported that in adult human peripheral blood, the concentrations of propionate and butyrate were estimated to be 14.4 ± 3.4 μmol/L and 15.1 ± 0.6 μmol/L, respectively (Zhao et al., 2007). Although these SCFAs gain access to the central nervous system via monocarboxylate transporter uptake in the cerebrovascular endothelium (Conn et al., 1983), they are rapidly metabolized to other compounds like ketones and long-chain fatty acids (den Besten et al., 2013). As reported by Hsiao et al. (2013), many other microbial metabolites besides SCFAs play important roles in brain development and behavior; further studies are required to confirm their effects in Chinese autistic children.

Our study had several strengths, including a large sample size, a rich data set that allowed us to control for many potential confounders, and the measurement of a broad spectrum of SCFAs. There were also several limitations to our study. Firstly, fecal SCFA data were only available from a single measurement; such measurements often vary and do not accurately reflect long-term bodily SCFA concentrations. Secondly, the levels of fecal SCFAs can appear too low in subjects eating high amounts of fiber owing to the fact that fiber not only generates SCFAs but also increases fecal bulk. It would have been better if all feces had been collected over 24 h, for example, to be able to see the total production. Thirdly, since more than 95% of the produced SCFAs are rapidly absorbed by the colonocytes, measurements of fecal SCFA concentrations reflect just the 5% of the produced SCFAs left unabsorbed (den Besten et al., 2013). SCFAs were only measured in feces, not in serum, and these may not represent absorbed SCFAs. Sakata suggested that fecal SCFA concentration should not be used as a measure of SCFA production or absorption (Sakata, 2019). Therefore, the conclusions in our study should be adopted with some caution. Fourthly, we did not collect information including pre- or perinatal infection, hospitalization, or early antibiotic or probiotic exposure, which might alter the gut microbiota and have been suggested to be potential risk factors for ASD (Penders et al., 2006). Fifth, the effects of dietary factors on the microbiome and the production of SCFAs always vary according to substrate source, e.g., wheats vs. inulins, omega 3s vs. *trans* fats, but it was very difficult to collect this information by dietary recall. Also, we could not rule out the impacts of the propionate added to many refined foods. Thus, residual and unmeasured confounding was still unavoidable due to measurement errors and/or missing adjustments for some unmeasured factors. Lastly, animal models showed that prenatal exposure to elevated levels of propionic acid at key developmental time periods had long lasting developmental effects (Foley et al., 2014). Further longitudinal studies are needed to elucidate the impact of total and different types of SCFAs at different periods on the occurrence of ASD.

In summary, this initial study in Chinese children aged 6–9 years old did not support the hypothesis that fecal SCFA levels might correlate with the presence of ASD. Future longitudinal studies with repeated assessment of other metabolic markers for SCFA (cecum, blood, cerebrospinal fluid, genetic partial metabolizer, etc.) are required to verify our findings.

## Data Availability Statement

All datasets generated for this study are included in the article/Supplementary Material.

## Ethics Statement

The Ethical Committee of the School of Public Health, Sun Yat-sen University approved this study (No. 2018047). The study was performed in accordance with the Helsinki Declaration as previously described (Ma et al., 2019). The parents or guardians of all subjects provided their written informed consent before the study.

## Author Contributions

JW and ZZ analyzed the data and wrote the manuscript. JW, HC, YL, WA, JL, and BM contributed to the data collection. LM and XC revised the manuscript. ZZ designed the project and supervised the study. JP assisted with SCFAs measurements. All authors have read and approved the manuscript.

## Conflict of Interest

The authors declare that the research was conducted in the absence of any commercial or financial relationships that could be construed as a potential conflict of interest.
