# Supplementary material for: Fecal Short-Chain Fatty Acids Levels Were Not Associated With Autism Spectrum Disorders in Chinese Children: A Case–Control Study
Source: Front Neurosci. 2019 Nov 29;13:1216. doi: 10.3389/fnins.2019.01216 (PMC6895143; doi:10.3389/fnins.2019.01216)
Supplement: Supplementary file 2 [file Image_1.pdf]

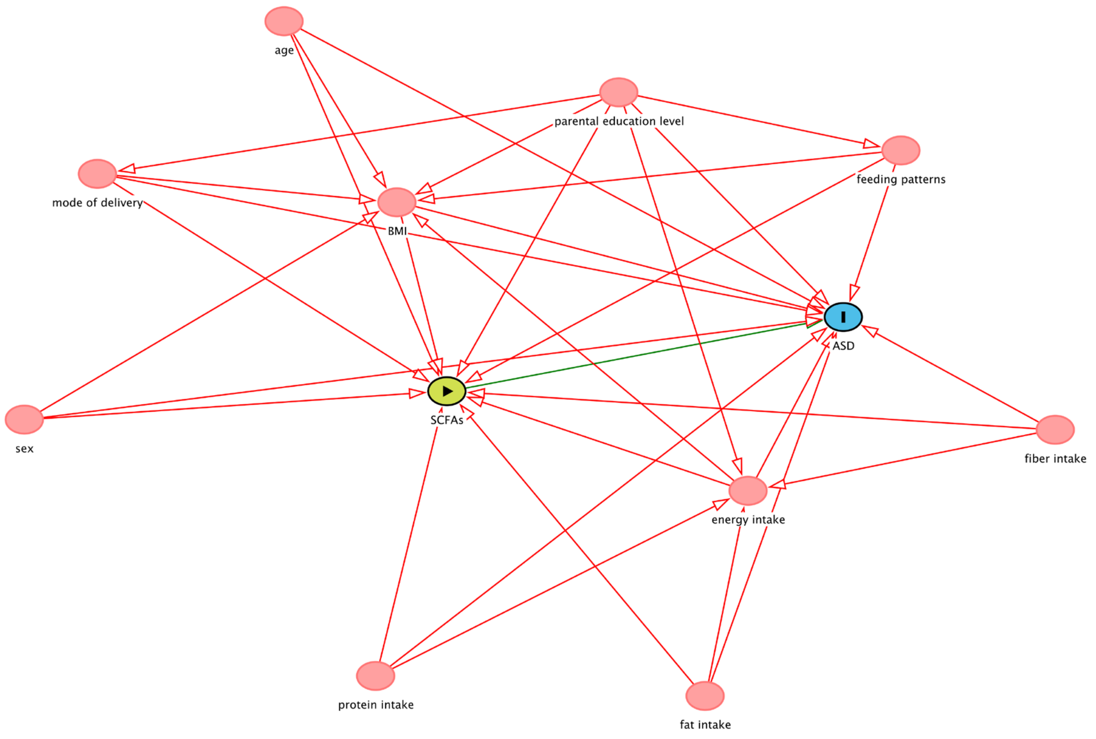

Figure S1: Directed acyclic graph illustrating relationship of short chain fatty acids and ASD of children, and confounders. SCFAs: short chain fatty acids; ASD: autism spectrum disorder.
